# Supplementary material for: Periodontal Disease in Young Adults as a Risk Factor for Subclinical Atherosclerosis: A Clinical, Biochemical and Immunological Study
Source: J Clin Med. 2023 Mar 12;12(6):2197. doi: 10.3390/jcm12062197 (PMC10051366; doi:10.3390/jcm12062197)
Supplement: Supplementary file 1 [file jcm-12-02197-s001.zip › jcm-2196903-supplementary.pdf]

Supplementary Materials

Serum¤ **GCF¤**

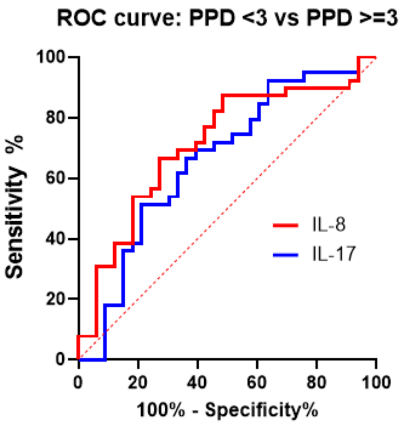

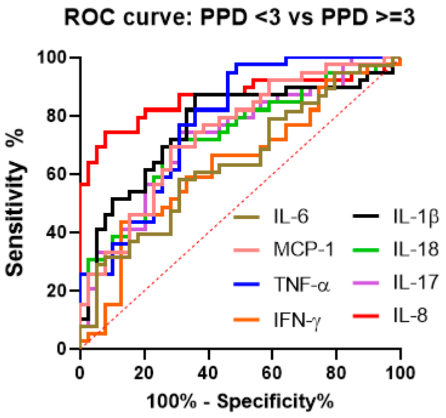

Supplementary Figure S1. Receiver operating characteristic curve (ROC) of cytokines in serum and GCF in discriminating PD according to PPD

Supplementary Table S1. Areas under the curves (AUC), 95% CI, the optimal cut-off values, sensitivity, and specificity of cytokines in serum and GCF in discriminating PD according to PPD (> or <)

**Parameter**	**AUC**	**95% CI**	***p****	***Cut-off****	***Sensitivity***	***Specificity (%)***
IL-8 (S)	0.73	0.60 to 0.83	0.0018	14.76	66.67	72.73
IL-17 (S)	0.67	0.54 to 0.79	0.0153	60.85	66.67	63.64
MCP-1 (GCF)	0.76	0.65 to 0.86	<0.0001	12.05	61.54	71.79
IL-6 (GCF)	0.64	0.52 to 0.76	0.0332	19.65	60.53	64.1
TNF-α (GCF)	0.78	0.68 to 0.88	<0.0001	9.6	76.92	66.67
IFN-γ (GCF)	0.64	0.511 to 0.76	0.0391	463.8	58.79	61.54
IL-1β (GCF)	0.77	0.66 to 0.89	<0.0001	403.6	71.79	71.79
IL18 (GCF)	0.72	0.61 to 0.83	0.0007	30	71.79	71.79
IL17 (GCF)	0.72	0.61 to 0.84	0.0006	66.05	71.79	69.23
IL-8 (GCF)	0.86	0.77 to 0.95	<0.0001	1859	82.05	79.49

AUC = Area under the curve; CI = Confidence interval *Cut-off values were calculated as the Index of Union.
